# Paget’s disease derived in situ from reserve cell hyperplasia, squamous metaplasia, and squamous cell carcinoma of the esophagogastric junction: a case report

**DOI:** 10.1186/s40792-018-0489-1

**Published:** 2018-07-25

**Authors:** Akihiko Sano, Shinji Sakurai, Chika Komine, Yuichi Tabe, Kana Saito, Takaharu Fukasawa, Shinsuke Kiriyama, Hideki Yamamoto, Masachika Tani, Hiroshi Naitoh, Ken Shirabe, Hiroyuki Kuwano

**Affiliations:** 1 Department of Surgery, Japan Community Healthcare Organization Gunma Central Hospital, 1-7-13 Kouncho, Maebashi, Gunma 371-0025 Japan; 2 Department of Diagnostic Pathology, Japan Community Healthcare Organization Gunma Central Hospital, 1-7-13 Kouncho, Maebashi, Gunma 371-0025 Japan; 3 Department of General Surgical Science, Gunma University Graduate School of Medicine, 3-39-22 Showa-machi, Maebashi, Gunma 371-8511 Japan

**Keywords:** Paget’s disease, Esophagogastric junction, Reserve cell hyperplasia, Squamous metaplasia

## Abstract

**Background:**

Extramammary Paget’s disease (EMPD) of the esophagus is a rare tumor, with most cases originating from invasive adenocarcinoma of the esophagus. Pure esophageal Paget’s disease, in which no underlying invasive carcinoma component is present, is extremely rare. In this report, we describe a case of EMPD of the esophagogastric junction with no evidence of invasive carcinoma.

**Case presentation:**

An 81-year-old Japanese woman with a 2-week history of abdominal distension presented to our hospital for assessment. Endoscopic examination revealed a mild elevated granular lesion, with a slightly depressed irregular mucosa, in the distal esophagus, with EMPD confirmed by biopsy. Thoracoscopic esophagectomy with lymph node dissection was performed, with Paget cells observed on microscopic examination in the lower part of the esophageal epithelium. Only a few Paget cells stained positively for PAS/Alcian blue. Immunohistochemically, negative staining for CK5 and p63 were identified in the Paget cells, with positive staining for CK7. Furthermore, an intraepithelial squamous cell carcinoma, with squamous metaplasia and reserve cell hyperplasia, was observed in the gastric mucosa of the esophagogastric junction, adjacent to the Paget cells.

**Conclusions:**

EMPD of the esophagus is a rare disease. We report a case of EMPD that was probably derived from a gastric squamous cell carcinoma, with squamous cell metaplasia and reserve cell hyperplasia, in the esophagogastric junction, which, to our knowledge, is the first report of this type of EMPD in the clinical literature.

## Background

Paget’s disease, defined as an intraepithelial invasion by a malignant glandular epithelial tumor, was first reported in the nipple and areolar skin [1], showing glandular differentiation on mucin histochemical analysis and/or immunohistochemical staining [2]. These cells appear organized in groups, with a nest-like pattern or gland-like structure, being preferably located in the epidermal basal layer [3]. Extramammary Paget’s disease (EMPD) has also been reported, typically occurring in skin areas with apocrine glands, with the most common sites being the vulva (65% of cases), the perianal region (20%), male genitalia (14%), and the apocrine gland-rich skin of the axilla [3, 4]. In most cases, EMPD is an intraepithelial lesion that is not associated with any underlying or distant cancers. However, there has been a report of EMPD linked to underlying adenocarcinomas of the vulva, vagina, cervix and corpus uteri, bladder, ovary, gallbladder, liver, breast, colon, and rectum [3], with these lesions being diagnosed as a pagetoid spread or growth of a carcinoma.

Several cases of esophageal EMPD have been reported, with most cases classified as pagetoid growths originating from invasive squamous cell carcinoma, adenosquamous cell carcinoma, or adenocarcinoma [5]. A pure esophageal Paget’s disease, in which no underlying invasive carcinoma component is present, is extremely rare. In this report, we describe the unique case of esophageal Paget’s disease, probably derived from a squamous cell carcinoma in situ, with reserve cell hyperplasia and squamous metaplasia of the gastric mucosa, in the esophagogastric junction.

## Case presentation

An 81-year-old Japanese woman with a 2-week history of abdominal distension presented to our hospital for assessment. The patient did not have a past history of malignancy, with only a cesarean section as a relevant feature in her history. Endoscopic examination at a previous hospital revealed the presence of early carcinomas in the stomach and distal esophagus. The patient was referred to our hospital for endoscopic resection.

**Fig. 1:**
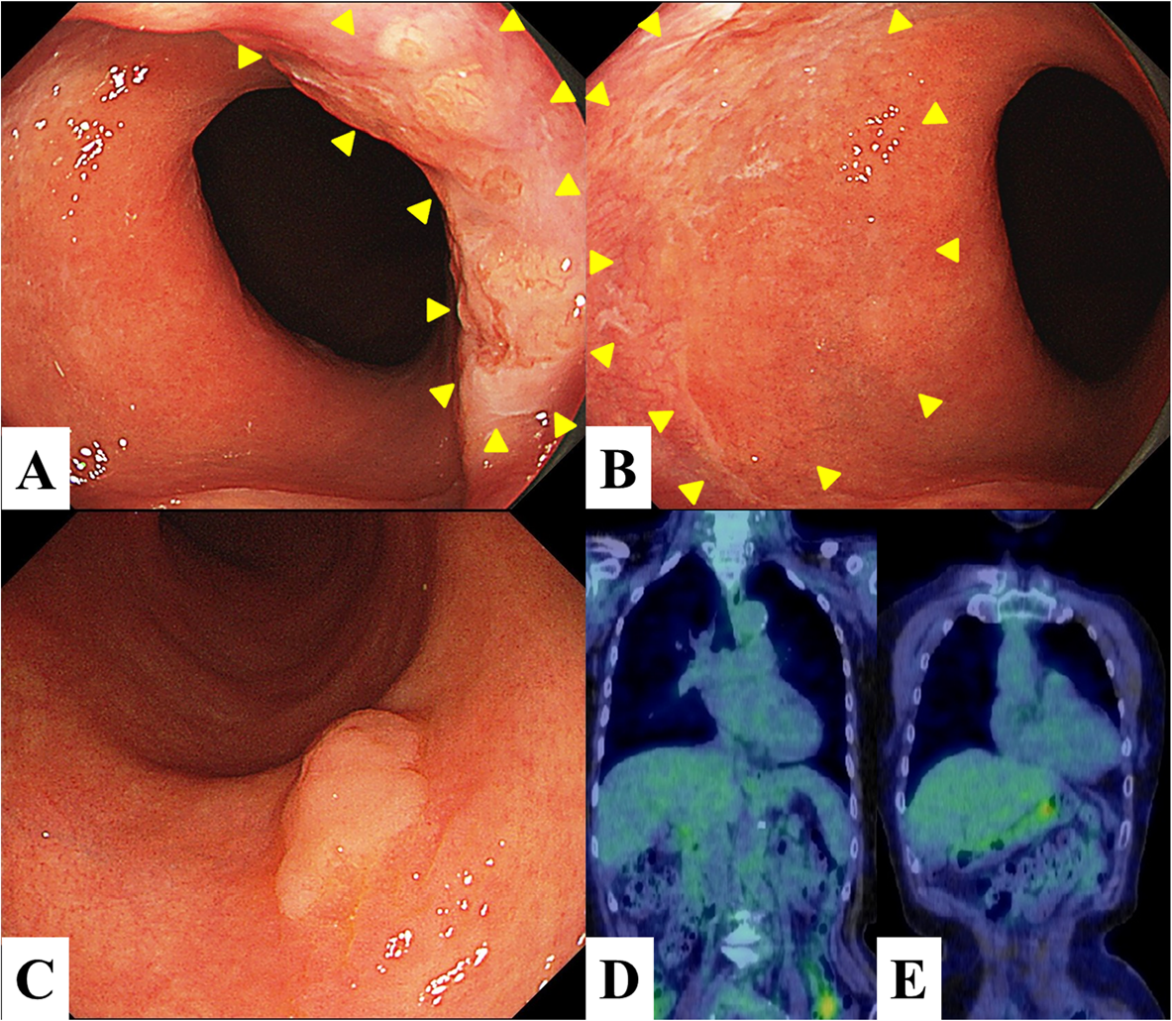
Endoscopy and FDG-PET/CT findings. **a**, **b** Endoscopy findings, showing a mild granular elevated lesion with slightly depressed irregular mucosa extending from the anterior wall to the right wall of the distal esophagus (arrowheads). **c** A gastric superficial elevated-type tumor, located in the lower body of the stomach. **d** FDG-PET/CT showing no significant FDG accumulation in the distal esophagus, nor in any other organs. **e** Increase in FDG accumulation in the lower body of the stomach

Laboratory data, as well as serum carcinoembryonic antigen, squamous cell carcinoma antigen, and cytokeratin-19 fragment levels, were close to normal limits. Endoscopic examination revealed mild granular elevated lesions, with slightly depressed irregular mucosa, extending from the anterior wall to the right wall of the distal esophagus (Fig. 1a). This irregular mucosa further extended from the anterior wall to the left wall, with the boundary on the oral side being unclear (Fig. 1b). A superficial elevated tumor-like lesion was also observed in the lower body of the stomach, with a diameter of about 10 mm (Fig. 1c). Based on the endoscopic biopsy specimen, this gastric lesion was diagnosed as a well-differentiated tubular adenocarcinoma. On the other hand, the preoperative biopsy specimens of the esophageal tumor showed intraepithelial tumor cells, which were isolated or in clusters, and consisted of large clear cells with atypical nuclei and prominent nucleoli. No glandular structures and no obvious intracytoplasmic mucin were observed. These histological findings were consistent with a malignant melanoma, with a pagetoid spread of invasive adenocarcinoma or squamous cell carcinoma, and Paget’s disease as a differential diagnosis. Immunohistochemically, the tumor cells diffusely stained positive for CK7 and partially for CK20, with negative staining for S100 protein and HMB-47. On the basis of these results, a diagnosis of malignant melanoma was excluded. All human mucin core proteins examined (MUC2, MUC5AC, and HIK1083) were also negative. Furthermore, p53 overexpression was observed in all tumor cells. From these results, we diagnosed the tumor as Paget’s disease or a pagetoid spread of an esophageal carcinoma. On enhanced computed tomography (CT) and [18F]-fluoro-deoxy-glucose positron emission tomography (FDG-PET)/CT imaging, no lymph node and distant metastases were identified (Fig. 1d). FDG uptake was observed only in the lower body of the stomach, with these lesions considered to reflect past endoscopic submucosal dissection (ESD) for early gastric cancer (Fig. 1e). Although we could not define the margin of the tumor, previous reports of esophageal Paget’s disease indicated a wide extension of Paget cells in the esophageal mucosa. On the basis of these findings, we planned ESD for the treatment of the gastric lesion, followed by a thoracoscopic esophagectomy (TE) and hand-assisted laparoscopic proximal gastrectomy (HALPG) for the treatment of esophagogastric Paget’s disease. Histological examination of the ESD specimen revealed a well-differentiated mucosal adenocarcinoma (11 mm × 8 mm) without lymphovascular involvement. The lateral and vertical margins of the resected tissue were free of tumor cells, and ESD was considered as a curative resection.

TE and HALPG, with lymph node dissection, were performed at 43 days after the gastric ESD. Regional lymph nodes were dissected, with no metastatic invasion identified in the thoracic and abdominal lymph nodes. Reconstruction with a gastric tube was performed after esophagectomy, using a hand-assisted laparoscopy procedure via a post-sternal route.

**Fig. 2:**
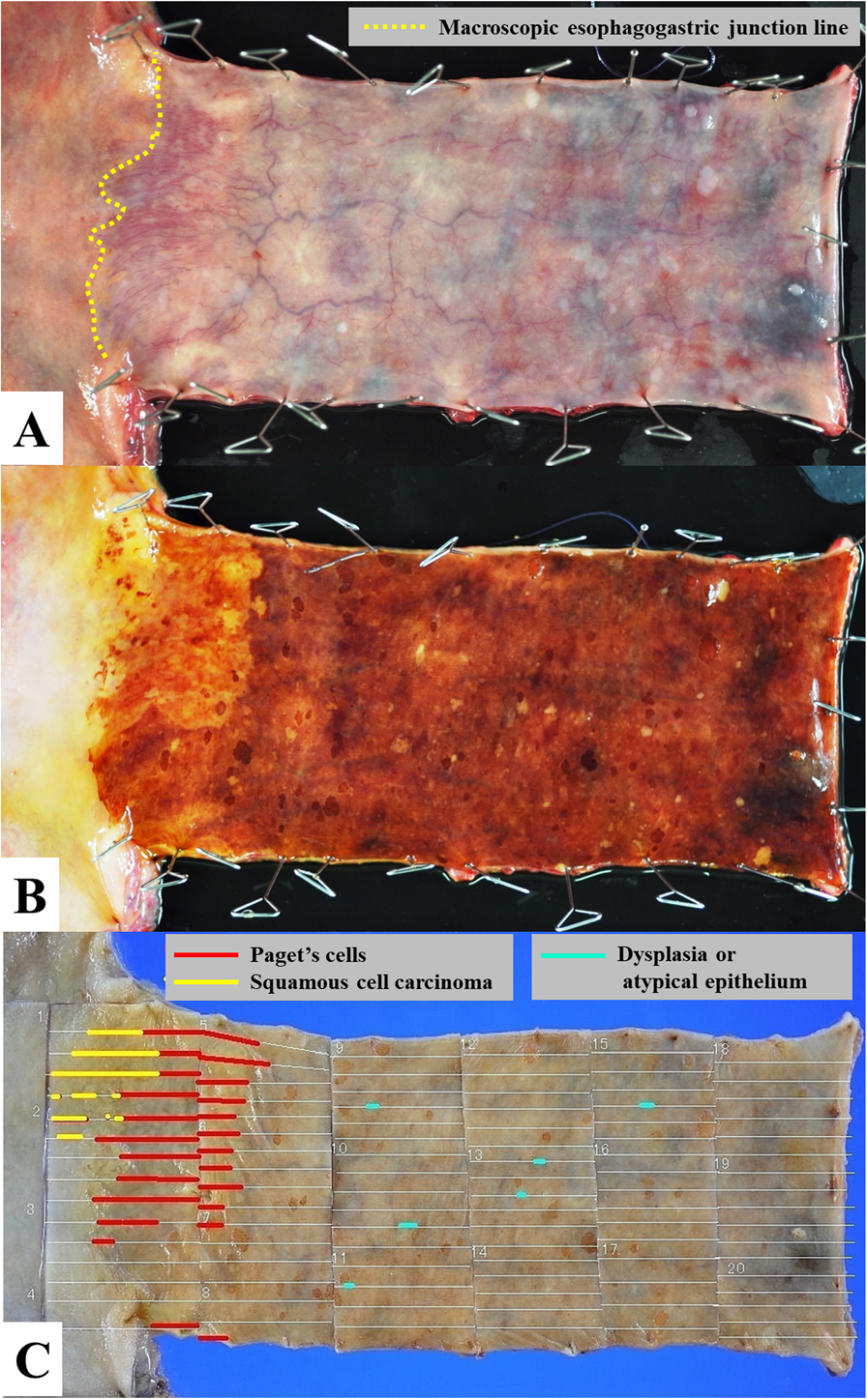
Surgically resected specimen of the lesions in the esophagus and proximal stomach. **a** Macroscopic finding of the specimen, showing a slightly irregular elevated lesion with slightly depressed mucosa in the lower thoracic and abdominal esophagus. **b** Iodine-unstained area of the distal esophagus and an isolated iodine-stain area in the gastric mucosa of the esophagogastric junction. **c** Schematic representation of the distribution of Paget cells and squamous cell carcinoma of the esophagogastric junction. The colors used represent the following: red, Paget cells; yellow, squamous cell carcinoma; blue, dysplasia or atypical epithelium

**Fig. 3:**
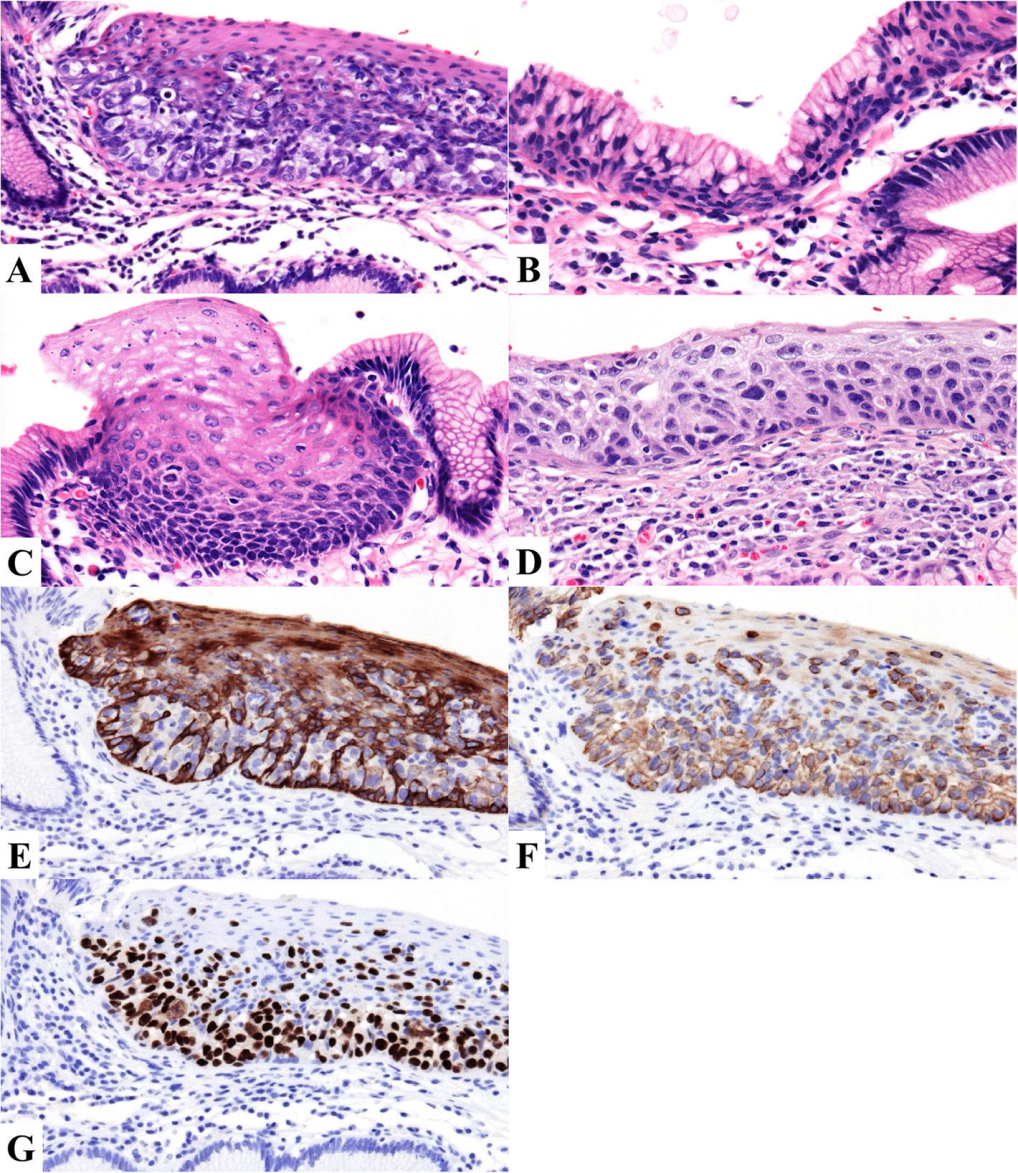
Microscopic findings. **a** Hematoxylin and eosin staining of a tumor section, showing neoplastic cells (Paget cells) with a large nucleus and a pale-staining cytoplasm in the lower part of the esophageal epithelium. **b**, **c** Reserve cell hyperplasia (**b**) and squamous metaplasia (**c**) in the esophagogastric junction. **d** A squamous cell carcinoma component identified in the same area. **e**–**g** Immunohistochemical staining of Paget cells. CK5 expression was not detected in Paget cells (**e**). CK7 (**f**) and p53 expression (**g**) were observed

Histological examination of the surgically resected specimen was performed. Macroscopically, the mucosa of the lower thoracic and abdominal esophagus was slightly irregular and depressed, with submucosal capillary hyperplasia (Fig. 2a). No tumor mass or ulceration was observable in the resected material. With iodine staining, the mucosa of the lower esophagus, which was congruous with the irregular and depressed area, did not stain. Furthermore, isolated small iodine-stained foci were observed in the gastric mucosa adjacent to esophagogastric junction (Fig. 2b). Microscopically, these foci consisted of squamous metaplasia of the gastric mucosa. The sectioned tissues were stained with hematoxylin and eosin (HE) and periodic acid-Schiff (PAS)/Alcian blue. As well, immunohistochemical staining for CK5, CK7, CK20, CDX2, MUC2, MUC5AC, HIK1083, p53, p63, S100, and HMB-45 was performed. Microscopic examination revealed neoplastic cells, with a large atypical nucleus and pale-staining cytoplasm, in the lower part of the esophageal epithelium, occurring either singly or in clusters (Fig. 3a). Reserve cell hyperplasia (Fig. 3b) and squamous metaplasia (Fig. 3c) were observed in the gastric mucosa, adjacent to the esophagogastric junction, and an intraepithelial squamous cell carcinoma (SCC) was observed within the squamous metaplasia (Fig. 3d). Components of the intraepithelial squamous cell carcinoma were identified following the Paget cells in the esophageal squamous epithelium. Only a few Paget cells stained positively for PAS/Alcian blue. Immunohistochemically, negative staining for CK5 (Fig. 3e) and p63 was identified in Paget cells, with positive staining for CK7 (Fig. 3f). The Paget cells showed no reactivity for intestinal mucin (MUC2) and gastric foveolar mucin (MUC5AC), but a few Paget cells were positive for gastric gland mucin (HIK1083). On the other hand, the intraepithelial SCC showed positive reactivity for CK5 and p63, but no reactivity for CK7 and CK20. Overexpression of p53 was observed in both Paget cells (Fig. 3g) and the intraepithelial SCC. Histochemical and immunohistochemical results are summarized in Table 1, and schematic representation of the distribution of Paget cells and squamous cell carcinoma of the esophagogastric junction is shown in Fig. 2c. Because there were any findings of Barrett’s esophagus neither endoscopically nor pathologically, macroscopic esophagogastric junction and pathological squamocolumnar junction were identical. Regional lymph node metastases were not identified on pathological assessment.

**Table 1:** Result of histochemical and immunohisotochemical examinations in Paget cell and squamous cell carcinoma (SCC) components

Methods	Paget cell component	SCC component
PAS/Alcian blue	+ (partial)	−
CK5	−	+
CK7	+	−
CK20	+ (partial)	−
CDX2	−	−
MUC2	−	−
MUC5AC	−	−
HIK1083	+ (partial)	−
p53	+	+
p63	−	+
S100	−	−
HMB-45	−	−

At the last follow-up, conducted 2 years and 8 months after surgery, the patient’s health status was fairly good, with no recurrence of the EMPD or carcinoma.

### Discussion

EMPD was first described in a patient with urinary bladder carcinoma in 1889 [3]. Since this initial report, EMPD has been described in various sites of the body, most commonly the vulva, perianal region, scrotum, penis, and axilla [6]. EMPD is subdivided into primary and secondary types on the basis of the presence or absence of associated malignancies. Primary EMPD is thought to be derived from an underlying neoplastic transformation of the intraepidermal portion of a sweat gland, whereas secondary EMPD is caused by the intraepidermal spread of neoplastic cells, typically derived from an underlying adenocarcinoma [7, 8].

Esophageal Paget’s disease is quite rare, with only a few cases having been reported [9–13]. Yates and Koss [9] described esophageal Paget’s disease associated with a poorly differentiated squamous cell carcinoma of the distal esophagus, whereas Norihisa et al. [10] reported a case of adenosquamous carcinoma of the esophagus with pagetoid extension of the adenocarcinoma component. Therefore, both of these cases were diagnosed as a pagetoid growth of an advanced esophageal carcinoma. On the other hand, Nonomura et al. [11] and Matsukuma et al. [12] reported esophageal Paget’s disease associated with an early underlying carcinoma, one being an intraepithelial carcinoma and the other, a minimally invasive adenocarcinoma of the esophagus. Ishihara et al. [13] also reported a case of an early invasive carcinoma, which consisted of pagetoid squamous cell carcinoma in situ combined with early invasive components and choriocarcinoma at the metastatic site. Abraham et al. [5] reported a close relationship between Paget cells in the esophagus and an underlying poorly differentiated adenocarcinoma in the esophagus or esophagogastric junction. From these reports, all previously reported cases of Paget’s disease of the esophagus were thought to be secondary to an underlying carcinoma, although the malignant component varied in each case. In our case, we identified an SCC component, with squamous metaplasia and reserve cell hyperplasia, in the gastric mucosa of the esophagogastric junction, which was followed by Paget cells. However, unlike typical Paget’s disease, only few Paget cells were positive for PAS/Alcian blue staining and immunohistochemically positive for gastric gland mucin, whereas a strong p53 overexpression was observed in both SCC component and Paget cells.

Reserve cells are small undifferentiated cells found as a single layer beneath the endocervical columnar epithelium. They have the capacity to transform into both endocervical columnar and squamous epithelium in the endocervix [14]. Reserve cell hyperplasia and epithelial dysplasia are frequently observed in the squamocolumnar junction of the cervix uteri, and squamous cell carcinoma of the cervix uteri is considered to be derived from these changes [15]. Reserve cell hyperplasia and squamous cell metaplasia of the gastric mucosa are rare phenomena. In 1981, Takubo [15] reported the resemblance of squamous cell metaplasia to reserve cell hyperplasia in the cervix uteri and considered squamous metaplasia or reserve cell hyperplasia with atypical change as a precursor of SCC in the esophagogastric junction. However, this hypothesis has not been fully elucidated. From histological findings and immunohistochemical results in our case, we speculate that the Paget cells were derived from the squamous cell carcinoma, developing in the squamous metaplasia and reserve cell hyperplasia of the esophagogastric junction. The difference in the pattern of expression of cytokeratin and p63 might reflect the glandular differentiation of tumor cells.

FDG-PET/CT imaging is currently accepted as the most accurate technique for exploring metastatic lesions of a solid tumor. The combination of metabolic and structural information provided by the PET and CT portions, respectively, has improved the accuracy of tumor staging, detection of recurrence, and therapeutic monitoring, having an enormous impact on patient management [16, 17]. In patients with EMPD, 18F-FDG PET/CT diagnosis of primary lesions is mainly dependent on the thickness of the lesions, whereas it is more sensitive for the diagnosis of lymph node and distant metastases [18]. In this case, thick primary lesions showed an intense uptake of 18F-FDG (SUVmax 14.9 and 7.5), whereas thin primary lesions showed only a mild 18F-FDG uptake (mean SUVmax 3.25 ± 0.24). Three of the 10 cases reported, however, showed no 18F-FDG uptake at primary site, as in our case. In 3 of these 10 cases with lymph node invasion and distant metastases of EMPD were upstaged by PET/CT, rather than conventional staging examination. To determine the appropriate treatment strategy for EMPD based on staging, PET/CT may play an important role, although some EMPD might be 18F-FDG negative.

Traditionally, EMPD has been surgically managed, especially in the early stage of the disease. Achieving adequate margins for the primary lesions is an important factor in reducing the risk of recurrence. In patients unfit for radical surgery, radiotherapy is proposed as alternative treatment, as long as invasive disease has been excluded [19]. In the surgical treatment of esophageal cancer, thoracoscopic esophagectomy is generally regarded, and accepted, as a minimally invasive surgery [20]. Biere et al. reported on the short-term benefits of minimally invasive esophagectomy for patients with resectable esophageal cancer, with prevention of pulmonary infection being an important benefit. Furthermore, thoracoscopic esophagectomy with three-field lymphadenectomy, pursuing best loco-regional control by surgery, is a feasible and safe alternative treatment commonly performed in Japan [21]. Our case was diagnosed as early stage Paget’s disease of the esophagus by endoscopic, CT, and PET/CT findings. But because of the unclear and extensive proximal margin of the tumor, a thoracoscopic esophagectomy was performed to obtain a wide local excision of the EMPD. However, in the pathological diagnosis, Paget’s disease and squamous cell carcinoma were identified in the mucosal layer. Therefore, curative resection with ESD could have been possible. ESD is an effective treatment for superficial esophageal neoplasms. Funakawa et al. [22] reported a success rate of 99.4% (164/165) for en bloc resection and 90.9% (150/165) for complete en bloc resection, with no instance of fatal complications. However, the reported incidence of esophageal strictures after ESD for near-circumferential or circumferential esophageal neoplasms is extremely high at 88–100% [23]. Post-ESD strictures seriously lower patients’ quality of life, being associated with several symptoms, including dysphagia, nausea, vomiting, weight loss, and even cachexia. In contrast, esophagogastric junction cancers have a high rate of submucosal invasion, irrespective of size, compared to non-junctional cancers [24]. Furthermore, the rates of positive lymphatic and/or venous invasion were remarkably higher in junctional cancers [24]. Therefore, when ESD is performed for near-circumferential junctional cancer as in our case, attention must be paid to the occurrence of esophageal stricture. It is important to evaluate the risk of recurrence by pathological diagnosis and to consider whether additional treatment, including surgical resection, should be performed.

## Conclusions

EMPD of the esophagus is a rare disease. We report a case of EMPD that was probably derived from a gastric squamous cell carcinoma, with squamous metaplasia and reserve cell hyperplasia, in the esophagogastric junction, which, to our knowledge, is a first description of this type of EMPD in the clinical literature.

## Abbreviations

CT: Computed tomography
EMPD: Extramammary Paget’s disease
ESD: Endoscopic submucosal dissection
FDG-PET: Fluoro-deoxy-glucose positron emission tomography
HALPG: Hand-assisted laparoscopic proximal gastrectomy
TE: Thoracoscopic esophagectomy
